# Legal Notes for Institutional Workers

**Published:** 1912-06-22

**Authors:** 


### LEGAL NOTES FOR INSTITUTIONAL WORKERS.

(The Editor will be pleased to answer Legal queries in this column.)

### The Importance of a Written Agreement.

The member of the junior staff who is about to accept the position of assistant to a doctor in general practice should pay attention to a case which was heard in the King's Bench Division a short time since. It demonstrates the importance not only of having a written agreement, but of having all the terms of that agreement carefully provided for. In the case in question a doctor who wanted an assistant wrote to the plaintiff, offering to give him a salary of £200 a year and to let him have the use of a house rent free. The letter also set forth the plaintiff's duties; but nothing was said as to the date when the employment was to commence. The plaintiff did, in fact, commence to work pursuant to the agreement, but circumstances arose which rendered it necessary for him to bring an action for breach of contract. It was held that, having regard to a statute which provides that certain contracts shall be in writing, there was here no contract which could be sued upon, inasmuch as the writing, such as it was, did not contain all the material terms. In the result, the plaintiff was non-suited. What is the moral? Not only should an agreement of this kind be put into writing, but care should be taken to make it embody all the terms. Everything may appear to be plain sailing and simple at the time of the contract; but no one can foresee all difficult questions which may arise in the future. Disputes having arisen between the parties it is satisfactory to have at least some of them settled by the terms of the contract.

### Refusal to Allow a Child to Undergo Operation.

What has taken place in Court when it is said that "a rule *nisi* for a *certiorari* was moved for"? Such a statement is, doubtless, as obscure to the medical man as the description of a simple ailment in medical language is to the lawyer. Under cover of this high-sounding phrase, however, the decision of a bench of magistrates in relation to an alleged offence under the Children Act, 1908, has recently been considered. The facts were very simple. A schoolgirl, aged five, was suffering from cleft palate. The medical officer was of opinion that her inability to articulate, which was interfering with her education, was due to this defect. He recommended a simple operation; but the child's father would not consent. He was therefore summoned under the Children Act, 1908, section 12 of which provides that "a parent or other person legally liable to maintain a child or young person shall be deemed to have neglected him in a manner likely to cause injury to his health if he fails to provide . . . medical aid, or if, being unable otherwise to provide such . . . medical aid, he fails to take steps to procure the same to be provided under the Acts relating to the relief of the poor." The justices convicted, and steps were taken to set aside the conviction. In giving judgment the Lord Chief Justice said: "The question before the justices was simply one of fact—namely, whether the applicant had neglected to provide medical aid for the child. The question on the merits might be properly raised in another proceeding; but I am clear that *certiorari* does not lie in such a case." As other proceedings will probably ensue we refrain from comment upon this decision, which appears to be the first on record in relation to this important Act.

### The One-eyed Workman.

The problem of the man who has lost the sight of one eye is one of the most difficult which presents itself to those who are called upon to administer the Workmen's Compensation Act. In a case recently decided in the House of Lords it has been held that in settling whether and to what extent a one-eyed man is incapacitated, the County Court judge must have regard to the fact that a one-eyed man is *ex necessitate* less likely to obtain employment than a man whose vision is unimpaired. The Lord Chancellor laid it down that the true meaning of the statute is that "incapacity for work"—the term employed by the Act—includes the case of a workman's eligibility to obtain work being diminished or lost, or, in other words, of his capacity to get work being impaired or destroyed. Wholly apart, then, from any medical testimony to the effect that the sound eye will remain sound, and that for certain kinds of work a one-eyed man is quite efficient, compensation, it is reasonable to think, will probably be awarded.

### A Point for Medical Witnesses.

The medical witness who is called on behalf of the employer when the case of a one-eyed man is under consideration will inevitably be asked whether there is any danger of the other eye being injured. In a recent case at the Banbury County Court, the judge adopted what appears to us to be an excellent method of dealing with a case of this character. A farm labourer who had lost the sight of one eye, but who was able to continue his work, had entered into an agreement to compound all future claims for £50. The question was whether the amount was adequate. An eminent specialist, who gave evidence, was constrained to admit that so long as the old eye remained in the socket there was danger of inflammation which might spread to the other eye; but he added that the risk would be reduced to the vanishing point if the old eye were removed. In these circumstances the judge said that the employer should add something to the compensation in order to enable the man to undergo the necessary operation. Eventually £60 was awarded, it being left to the workman to decide whether he would undergo the operation or not.

